# Effects of low-pressure Valsalva maneuver on changes in cerebral arterial stiffness and pulse wave velocity

**DOI:** 10.1371/journal.pone.0308866

**Published:** 2024-09-27

**Authors:** Eun-Seon Yang, Ju-Yeon Jung, Chang-Ki Kang

**Affiliations:** 1 Department of Health Sciences and Technology, Gachon Advanced Institute for Health Sciences & Technology (GAIHST), Gachon University, Incheon, Republic of Korea; 2 Institute for Human Health and Science Convergence, Gachon University, Incheon, Republic of Korea; 3 Neuroscience Research Institute, Gachon University, Incheon, Republic of Korea; 4 Department of Radiological Science, College of Medical Science, Gachon University, Incheon, Republic of Korea; King Faisal University College of Veterinary Medicine and Animal Resources, SAUDI ARABIA

## Abstract

The Valsalva maneuver (VM), commonly used to assess cardiovascular and autonomic nervous system functions, can induce changes in hemodynamic function that may affect cerebral vascular functionality, such as arterial elasticity. This study aimed to investigate the effects of low-pressure VM on cerebral arterial stiffness and cerebral vascular dynamics. Thirty-one healthy young participants (average age 21.58±1.72 years) were recruited for this study. These participants were instructed to maintain an expiratory pressure of 30–35 mmHg for 15 seconds. We measured the vasoconstriction and vasodilation diameters (VCD and VDD) of the common carotid artery (CCA), as well as systolic and diastolic blood pressures (SBP and DBP), before and after VM (PRE_VM and POST_VM). Additionally, we assessed mean arterial pressure (MAP), pulse pressure (PP), pulse wave velocity (PWV), and arterial stiffness. Our findings revealed significant increases in both the VCD and VDD of the CCA (2.15%, *p* = 0.039 and 4.55%, *p*<0.001, respectively), MAP (1.67%, *p* = 0.049), and DBP (1.10%, *p* = 0.029) following low-pressure VM. SBP showed an increasing trend, but this was not statistically significant (*p* = 0.108). Interestingly, we observed significant decreases in arterial stiffness and PWV in POST_VM when comparing with PRE_VM (*p*<0.001 and *p*<0.001, respectively). In conclusion, our study demonstrated the effectiveness of low-pressure VM in reducing the PWV and stiffness of the CCA. This suggests that low-pressure VM can be a simple and cost-effective method to reduce cerebrovascular stiffness in a brief interval, without the need for specific environmental conditions.

## Introduction

Stroke is the second leading cause of death worldwide, and the importance of its prevention is rapidly increasing [1]. The cerebrovascular reserve (CVR) is a major regulatory capacity for the prevention of cerebrovascular diseases such as stroke [2]. CVR refers to the ability to maintain blood flow to the brain tissue as needed despite various environmental changes [3]. A decreased CVR induces a lack of blood supply compared to the metabolic demand required in the brain tissue. Therefore, this insufficient blood supply can result in an increased incidence of cerebrovascular disease [4]. Thus, CVR has been reported as a predictor of ischemic stroke, and sufficient CVR performance contributes to improved cerebral flow [5]. Incidentally, CVR can be influenced by vascular conditions such as arterial stiffness or hypertension [6, 7]. Arterial stiffness is the main cause of impaired vascular ability and decline in cognitive function [8, 9]. Therefore, it is important to increase or maintain the elasticity of the arteries to supply sufficient blood to tissues and prevent cerebrovascular diseases. However, proper management to control arterial stiffness is still not well known.

Previous studies have reported pharmacological approaches to control arterial stiffness; however, the potential side effects of these drug treatments in individuals are difficult to predict [10, 11]. In particular, vasodilators using nitrate compounds can cause headaches owing to nitric oxide secretion [12], and side effects such as bronchospasm, bradycardia, and flushing may also occur [13]. Additionally, an increase in drug dependence cannot be ruled out. To overcome these limitations, other methods include aerobic exercise training; however, these are not optimally focused on treating the changes in arterial stiffness [14]. Aerobic exercise requires an appropriate environment, time, and cost. Furthermore, quantitative evaluations and scientific evidence on the effect of aerobic exercise on cerebrovascular stiffness are scarce. Therefore, non-invasive and effective stimulation methods are needed to control vascular dynamic function and reduce arterial stiffness.

The Valsalva maneuver (VM) is a breathing method that promotes arterial circulation by increasing intrathoracic and abdominal pressures through forced exhalation with a closed glottis [15]. The baroreflex mechanism and autonomic nervous system are activated to regulate increased mean arterial pressure (MAP). In this mechanism, the baroreflex sends signals to the brain to increase heart rate (HR) and activate the sympathetic nervous system (SNS) to offset the reduced stroke volume [16, 17]. Briefly, the increased MAP by VM causes the cardiac output to decrease, which makes the baroreceptor activate the SNS to compensate for the blood flow reduction [18, 19]. In this mechanism, VM breathing pressure can modulate arterial pulsatility by altering the perfusion pressure. Mechanical restraint of dilation occurs at the beginning of the VM, which is associated with pulsatility reduction. Conversely, the relaxation phase of VM induces increased pulsatility and vasodilation through decreased perfusion pressure, which can affect blood vessels stiffness [20]. Therefore, VM can be used to directly alter the stiffness of the cerebral blood vessels. However, the existing VM requires an expiratory pressure of 40 mmHg or higher. The risks of performing such high-pressure VM, such as damage to blood vessel walls, have been widely reported [21]. Therefore, the application of existing VM as method of stiffness management is cautioned in elderly patients and those with hypertension [22]. Additionally, it is difficult to maintain high pressure even in healthy individuals. Thus, a modified VM method is required for the safe and effective regulation of stiffness.

This study aimed to develop a new low-pressure VM approach to relieve the arterial stiffness of the common carotid artery (CCA) vessels and to evaluate the following hypotheses regarding vascular dynamic function and elastic change: First, low-pressure VM is effective in changing the mean arterial pressure (MAP) and pulse pressure (PP) by changing BP. Second, the increase in pressure in the body cavity due to the VM increases blood vessel diameters and reduces stiffness and pulse wave velocity (PWV). Thus, low-pressure VM performance improves cerebral blood flow, and the results can be used to assess the hemodynamic function of the cerebral vessels and aid in the effective early diagnosis and prevention of various cerebrovascular diseases.

## Materials and methods

### Participants

An experienced researcher (Ju-Yeon Jung) performed the statistical analysis, and the sample size was calculated using G*Power 3.1.9.4, relying on data from an internal pilot study [23]. The effect size was calculated using the vasoconstriction (VC) diameter of common carotid artery before and after Valsalva maneuver (VM), because VC is substantially related to vascular stiffness. Based on the internal pilot study findings (n = 11), the average diameters of VC before and after VM were 5.94 ± 0.47 and 6.19 ± 0.46, respectively. Thus, the sample size was calculated as 30 in two-tailed test (effect size = 0.537, α error probability = 0.05, statistical power = 0.8). Considering a potential dropout rate of 10%, 3 more participants were recruited. Thirty-three healthy adults in their twenties participated in the study. All participants provided written informed consent before the experiment began, and the study was approved by the Institutional Review Board (IRB no. 1044396-202207-HR-146-01; Approval date: August 29, 2022) of the Gachon University Bioethics Committee and the World Health Organization International Clinical Trials Registry Platform (Clinical Research Information Service (CRIS) number: KCT0009172). The authors confirm that all ongoing and related trials for this intervention are registered. The participants were directly recruited by flyers posted in public places in Incheon, Republic of Korea from March 27, 2023 to May 1, 2023 and the experiment was conducted until May 12, 2023. No follow-up was performed, and all data were collected at Gachon University.

They were restricted from consuming supplements or caffeine that could affect the blood vessels or autonomic nervous system for 24 hours before the experiment. Participants with cardiovascular diseases such as cerebrovascular disease, hypertension, or hypotension were excluded. Before the experiment, the maximum expiratory pressure of each participant was measured using a digital pressure gauge (SBH, SUNGJI Tech, Korea) connected to a mouthpiece. Participants who could not maintain over 30 mmHg of expiratory pressure and had abnormal biometric indicators defined below were excluded.

**Fig 1:**
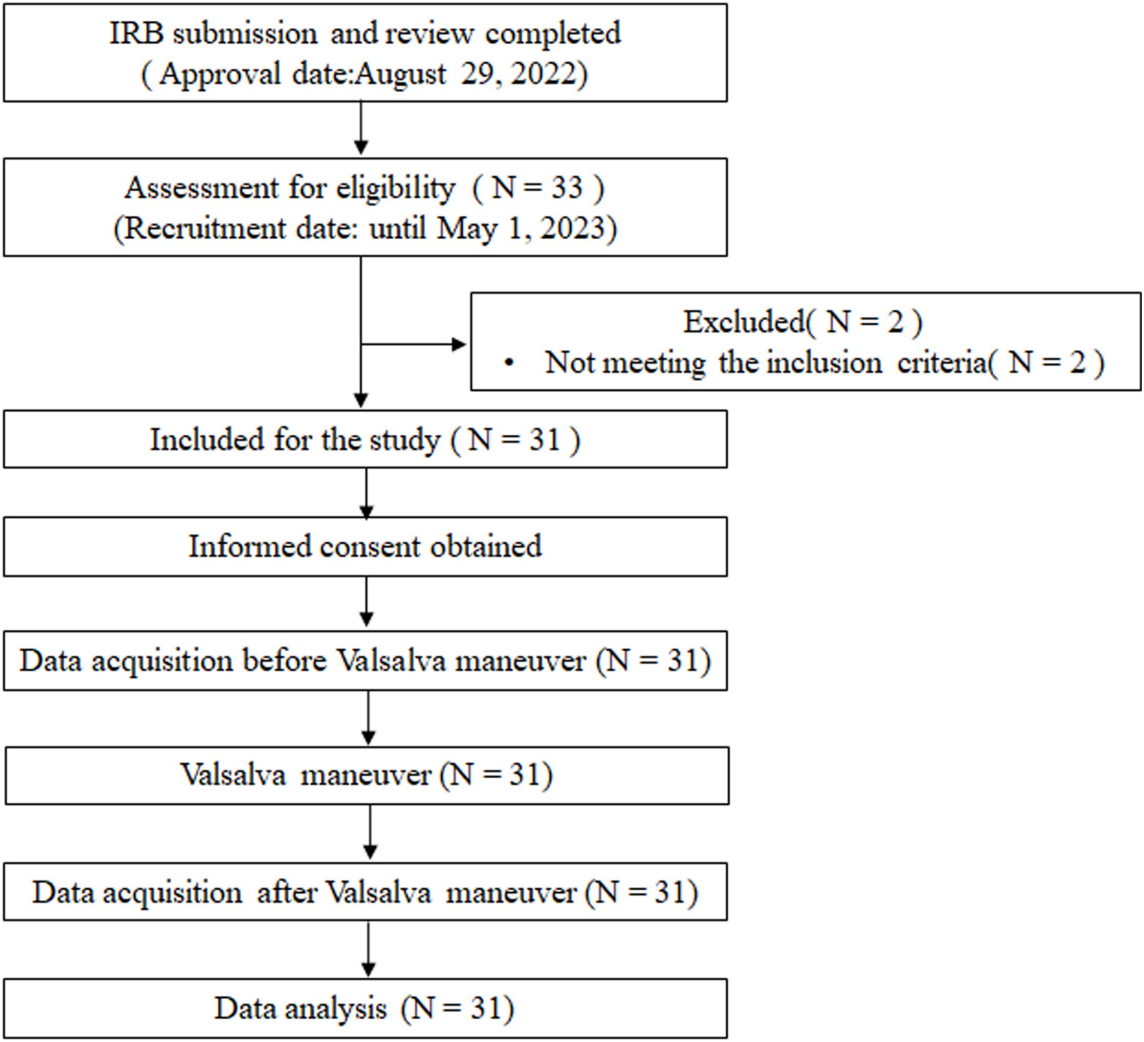
Flow chart of experimental protocol.

Normal adult BP range was defined as SBP and DBP < 120 and 80 mmHg, respectively [24]. However, when the PP was normal, an SBP of up to 130 mmHg was allowed [25]. HR of 60–90 beats per minute (bpm) [26], respiratory rate (RR) of 12–20 breaths per minute [27], and end-tidal carbon dioxide (EtCO2) of 30–40 mmHg [28] were defined as normal ranges. BP and HR were measured using a wearable smartwatch (Galaxy Watch 3, Samsung, Korea) that can measure BP without vascular compression. Other vital signs were measured using a patient monitoring system (BPM-770, Bionics, Korea). Consequently, two participants were excluded from the study. Finally, 31 participants (14 males and 17 females) were included in the study (Fig 1). Table 1 lists the personal characteristics and biosignals of the 31 participants.

**Table 1:** General characteristics and resting vital signs of the participants.

Variables	Total (n = 31)	Men (n = 14)	Women (n = 17)
Age (years)	21.58 ± 1.72	22.21 ± 2.19	21.05 ± 1.24
Height (cm)	167.90 ± 6.24	173.00 ± 4.31	163.70 ± 4.05
Weight (kg)	61.96 ± 9.30	68.71 ± 8.29	53.27 ± 5.82
AME BP (mmHg)	55.32 ± 19.22	68.36 ± 20.93	44.59 ± 8.13
SBP (mmHg)	114.74 ± 6.88	115.43 ± 6.81	114.72 ± 7.12
DBP (mmHg)	69.16 ± 4.98	67.43 ± 4.86	70.27 ± 4.78
HR (bpm)	71.48 ± 7.68	71.53 ± 8.81	71.35 ± 6.89
EtCO2 (mmHg)	41.33 ± 2.66	42.07± 2.30	40.69 ± 2.96
RR (breath/min)	17.60 ± 2.92	17.79 ± 2.19	17.43 ± 3.50

Abbreviations: AME, average maximum expiration; BP, blood pressure; DBP, diastolic blood pressure; EtCO2, end-tidal carbon dioxide; HR, heart rate; RR, respiratory rate; SBP, systolic blood pressure.

### Experimental protocol and task design

All experiments were conducted in the ultrasound room with the following procedures. The participants trained the VM using a 5.12-inch balloon in the supine position and were allowed to sufficiently practice for 3 min. A balloon is commonly used to perform Valsalva breathing. By exhaling air into the balloon, the Valsalva effect occurs, increasing the intra-abdominal pressure [29]. Once the participants were comfortable with VM breathing, their maximum expiratory pressure was measured using a digital pressure gauge connected to a mouthpiece. The participants were instructed to maintain their pressure between 30 and 35 mmHg and not exceed 35 mmHg during the VM.

**Fig 2:**
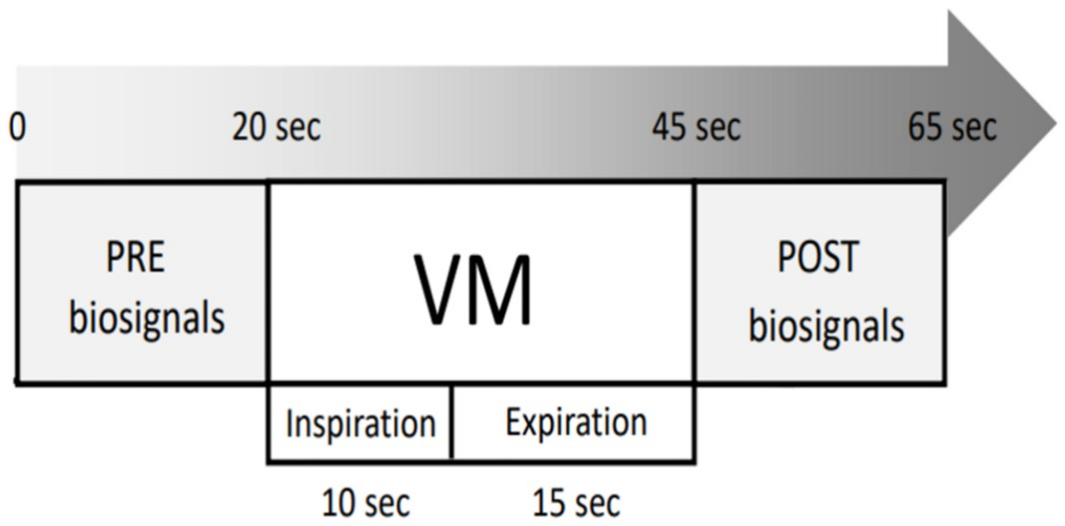
Biosignals acquisition protocols in pre and post VM. Biosignals represent the measurements of blood vessel diameter, blood pressure, and heart rate.

After pre-training, the participants took a break of approximately 5 min to allow the biosignals to return to the normal range before the experiment began. The experimental sequence was as follows: First, before performing the VM (pre-test), B-mode ultrasonography scan was performed for CCA vessel diameter and BP was simultaneously measured (Fig 2).

**Fig 3:**
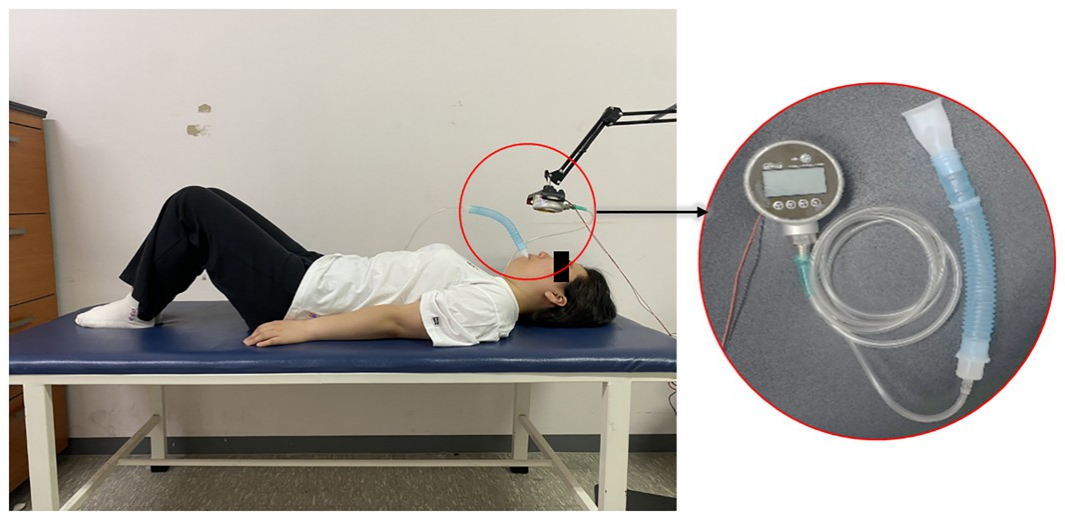
A representative participant performing a Valsalva maneuver while checking the real-time respiratory pressure indicator. A digital respiratory pressure meter connects to a mouthpiece with a tube.

Second, each participant performed the VM with an intensity of 30–35 mmHg. The single trial VM was performed as shown in Fig 3. The participants lay in a supine hook-lying position with both arms attached to their upper body and held the mouthpiece in their mouth. They then inhaled for approximately 10 s, and while their glottis was closed, the output value of the digital pressure gauge was adjusted to zero. The experimenter gave the subject a signal to start VM. They exhaled for 15 s after being instructed to begin the VM while watching the screen of the respiratory pressure gauge and were instructed to maintain the pressure between 30 and 35 mmHg as much as possible. At the end of the VM, a stop signal was given to the participants who slowly lowered their legs to return to supine.

Finally, after performing the VM (post-test), vital signs such as BP and CCA vessel diameter were measured within 20 s after VM. Transportation expenses were provided to increase participant compliance.

This experimental protocol was modified from the original IRB protocol because VM was chosen as a vascular stimulation method for healthy participants through pilot testing (N = 11). In addition, NIRS attached to the forehead was excluded due to difficulty in collecting accurate data and the effects of ‘during intervention’ could not be collected due to limitations of ultrasound measurements.

### Data acquisition and measurements

**Fig 4:**
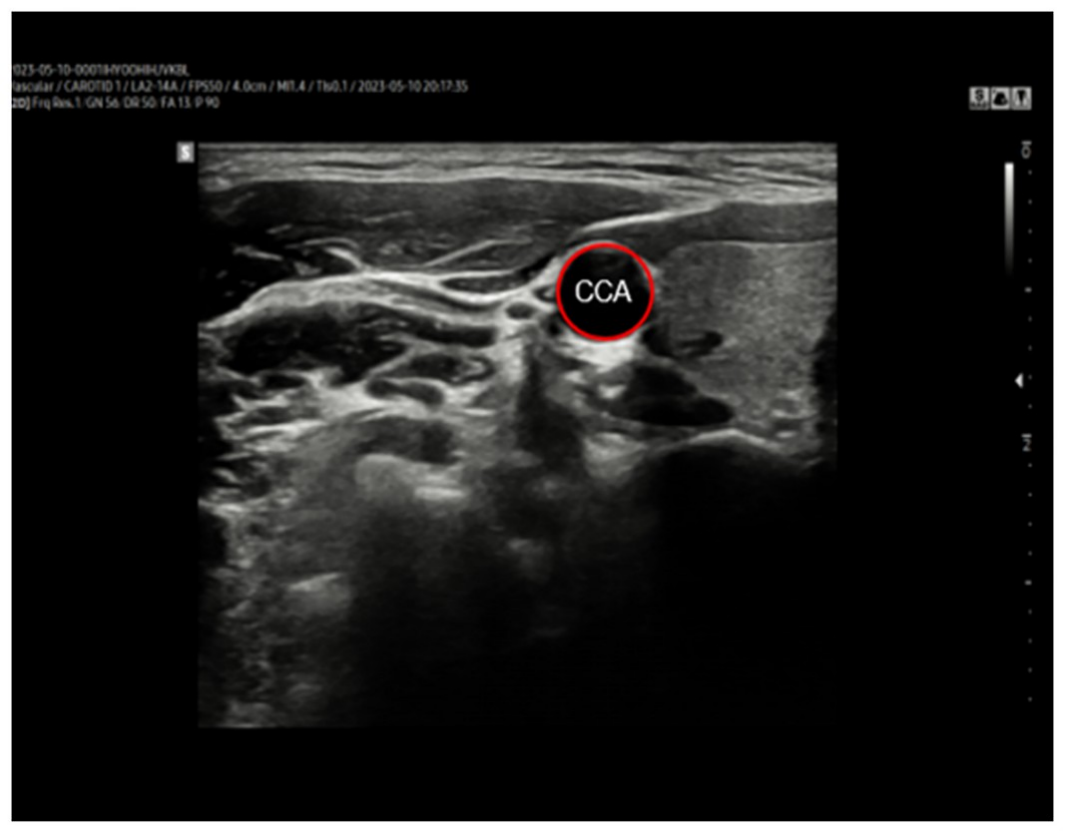
A representative ultrasound axis image. Abbreviation: CCA, common carotid artery.

To eliminate the experimenter bias, measurements and data analysis were performed by different researchers. All measurements were conducted by Ju-Yeon Jung who had substantial experience for ultrasonography. In this study, CCA vascular changes caused by the VM were assessed using Doppler ultrasound (RS85, Samsung, Korea), and the stiffness and PWV were calculated. The right side of the CCA was selected to collect CCA data before and after the VM performance. CCA diameter was measured using B-mode ultrasonography. To designate the location for CCA measurements, 2 cm below the CCA bifurcation was marked, and a cross-sectional image of the CCA was obtained by scanning for approximately 5 s (Fig 4). The diameters of vasoconstriction (VCD) and vasodilation (VDD) were measured based on the outer diameter of the CCA in digital imaging and communications in medicine (DICOM) images. The measurements were performed according to arterial measurement standards using the Radiant DICOM Viewer (Mediant, Poznan, Poland) [30].

BP was continuously and noninvasively measured using the photoplethysmography sensor of the smartwatch [31, 32]. The BP measurement took approximately 20 s and was collected before and after the VM performance. The measured data were automatically recorded using the Samsung Health Monitor application according to the manufacturer’s instructions.

### Calculations of PWV, stiffness, PP, and MAP

One of the factors of stiffness is a decrease in the elasticity of the blood vessels. This causes the walls of blood vessels to harden owing to the decrease in collagen fiber, making it difficult for arteries to deliver blood [33]. The stiffness index (β), which is the blood vessel tension, was calculated using the following formula with the diameters of CCA and the BP [34].


β=ln(SBP/DBP)×{VCD/(VDD-VCD)}

PWV is defined as the speed at which pressure waves generated in the cardiac systolic phase propagate along the arterial tree. It is an important clinical parameter for evaluating the risk of cardiovascular diseases and blood vessel conditions [35, 36]. PWV can be calculated using stiffness and blood density (ρ), using the following formula [37, 38].


$$PWV=\sqrt{\;\beta \;\times \frac{\left(DBP\times 0.13333\right)}{\left(2\times \rho \right)}}$$

ρ = density of blood (1.059 kg/m^3^).

PP and MAP were calculated using the measured BP. PP is a factor that identifies arteriosclerosis and has a strong correlation with stiffness. PP was calculated as the difference between SBP and DBP. MAP is the time-weighted average of the arterial pressure over the entire cardiac cycle [39]. The MAP was calculated using following formula [39].


$$MAP=DBP+\left(\frac{1}{3}\;\times PP\right)$$

### Statistical analysis

Data analysis was performed by Eun-Seon Yang. Statistical analyses were performed using the jamovi ver. 2.2.5 (The jamovi project (2021)). The Shapiro-Wilk test was conducted for the test of normality, and all parameters (except for pulse pressure) satisfied the normality. A paired sample t-test was used to analyze significant differences between the pre and post VM (PRE_VM and POST_VM) values. The difference between pre and post values was then calculated by subtraction (PRE_VM − POST_VM). To confirm the increase and/or decrease ratio, the rate of change was calculated from the difference between before and after VM execution.

To identify the correlation between the PWV, stiffness, and other related variables, a Pearson correlation analysis was conducted. The standard criterion of statistical significance (*p*<0.05) was applied to all analyses.

## Results

In this experiment, 33 individuals expressed their intention to participate, but 31 eligible participants met the inclusion criteria except for two persons. All participants successfully completed the procedures and assessments without any withdrawals or incidents, including accidents or side effects, during the measurements.

### PWV and stiffness

Low-pressure VM significantly reduced PWV (*p*<0.001). A reduction of 0.61 m/s in the PWV from PRE_VM (5.03 m/s) to POST_VM (4.41 m/s) was observed, and the reduction rate was 12.32%. Low-pressure VM also significantly reduced the stiffness (*p*<0.001). The POST_VM stiffness decreased by 1.51, from 6.17 to 4.67, and the reduction rate was 24.31%. These results show that low-pressure VM is effective in reducing the PWV and stiffness (Table 2).

**Table 2:** Vascular dynamic changes caused by VM performance.

Variables		Mean ± SD	ΔDIFF (Mean ± SD)	T	*p*
PWV (mm/s)	PRE	5.03 ± 0.86	0.61 ± 0.83	4.12	< .001*
	POST	4.41 ± 0.68			
Stiffness (β)	PRE	6.17 ± 2.22	1.51 ± 1.96	4.28	< .001*
	POST	4.67 ± 1.61			
SBP (mmHg)	PRE	111.65 ± 6.35	-1.52 ± 5.10	-1.66	0.108
	POST	113.16 ± 7.31			
DBP (mmHg)	PRE	67.39 ± 4.53	-1.29 ± 3.12	-2.30	0.029*
	POST	68.68 ± 6.07			
MAP (mmHg)	PRE	82.14 ± 3.12	-1.37 ± 3.71	-2.05	0.049*
	POST	83.51 ± 5.22			
PP (mmHg)	PRE	44.26 ± 8.85	-0.23 ± 2.49	-0.51	0.617
	POST	44.48 ± 8.25			
VCD (mm)	PRE	6.05 ± 0.50	-0.13 ± 0.33	-2.16	0.039*
	POST	6.18 ± 0.55			
VDD (mm)	PRE	6.60 ± 0.51	-0.31 ± 0.35	-4.89	< .001*
	POST	6.90 ± 0.61			

Δ is PRE_VM—POST_VM.

* < 0.05.

DBP, diastolic blood pressure; DIFF, difference value; MAP, mean arterial pressure; PP, pulse pressure; PWV, pulse wave velocity; SBP, systolic blood pressure; SD, standard deviation; VCD, blood vessel diameter during vasoconstriction; VDD, blood vessel diameter during vasodilation; VM, Valsalva maneuver.

### BP (SBP and DBP), MAP, and PP

BP increased after low-pressure VM was performed. The average SBP value increased by 1.52 mmHg, from 111.65 mmHg to 113.16 mmHg. This represented an increase of 1.35%; however, the difference was not statistically significant (*p* = 0.108). In contrast, DBP increased significantly after low-pressure VM (*p* = 0.029). The average DBP increased by 1.29 mmHg, from 67.39 mmHg to 68.68 mmHg, and the increase rate was 1.10%.

Similar significant results were obtained for the MAP, which was calculated using BP. The MAP significantly increased after low-pressure VM (*p* = 0.049), with an average increase of 1.37 mmHg and an increase rate of 1.67%, indicating that the pressure received by the blood vessels of the whole body for a cardiac cycle increased. However, there was no significant difference in the PP after low-pressure VM (*p* = 0.617). The difference between the PRE_PP and POST_PP was 0.23 mmHg (Table 2).

### VCD and VDD

After low-pressure VM, both systolic and diastolic blood vessel diameters increased significantly. The POST_VCD increased by 0.13 mm, from 6.05 to 6.18 mm (*p* = 0.039), reflecting a 2.15% increase. The POST_VDD increased by 0.31 mm, from 6.60 to 6.90 mm (*p*<0.001), reflecting a 4.55% change. The change rate of VDD was higher than that of VCD (Table 2).

### Correlation analysis of PWV and stiffness with variables

ΔVCD was significantly correlated with both ΔPWV and Δstiffness (Table 3). The average values of ΔVCD, ΔPWV, and Δstiffness were 0.13, 0.61, and 1.51, respectively. ΔVCD showed a significant positive correlation with ΔPWV (r = 0.49, *p* = 0.005) and Δstiffness (r = 0.51, *p* = 0.003). In contrast, ΔVDD was not significantly correlated both ΔPWV (r = -0.03, *p* = 0.890) and Δstiffness (r = 0.03, *p* = 0.886). ΔVDD had a weak negative correlation with ΔPWV. Therefore, the changes in PWV and stiffness increased significantly as the VCD diameter increased. Furthermore, ΔSBP, ΔDBP, ΔMAP, and ΔPP, which are variables related to BP, did not show any significant correlations with ΔPWV and Δstiffness.

**Table 3:** Comparison of the correlations of PWV and stiffness with variables.

Variables	ΔPWV		Δstiffness	
	r	*p*	r	*p*
ΔSBP	0.21	0.262	0.19	0.316
ΔDBP	0.27	0.139	0.23	0.214
ΔMAP	0.25	0.179	0.21	0.248
ΔPP	0.09	0.093	0.09	0.619
ΔVCD	0.49	0.005*	0.51	0.003*
ΔVDD	-0.03	0.890	0.03	0.886

Δ is PRE_VM—POST_VM.

* < 0.05.

DBP, diastolic blood pressure; MAP, mean arterial pressure; PP, pulse pressure; PWV, pulse wave velocity; SBP, systolic blood pressure; VCD, diameter of the blood vessel during vasoconstriction; VDD, blood vessel diameter during vasodilation.

## Discussion

We confirmed that low-pressure VM significantly improves the elasticity of the CCA. The proposed VM method uses an expiratory pressure of 30 to 35 mmHg, which is easier and safer to perform than the conventional VM method, in which an expiratory pressure of > 40 mmHg is required in clinical practice. In this study, low-pressure VM had a significant effect on increasing blood vessel diameter (VCD and VDD), increasing DBP and MAP, and reducing blood vessel stiffness (Table 2).

Although low-pressure VM increased SBP, the difference was not statistically significant (Table 2). However, a significant increase in MAP and DBP was observed. These results suggest that low-pressure VM provides sufficient intrathoracic pressure to increase the arterial blood pressure, such that 30–35 mmHg can induce a substantial change in arterial circulation.

In this study, the vascular diameters (VCD and VDD) increased significantly after low-pressure VM, and it is generally known that increased diameter results in decreased BP [40]. After low-pressure VM, however, increases in vessel diameter and BP were observed due to systolic overshoot caused by VM stimulation. In addition to the continued sympathetic activity in phase II of the VM mechanism, BP is excessively increased due to increased venous return and ventricular filling to restore BP in phase IV [41]. Furthermore, the diameter of the blood vessels can increase for BP recovery. According to the mechanism of VM, parasympathetic activity, which induces blood vessel relaxation, is reflexively increased in the final phase to offset the sympathetic effects activated in the early phase of VM [42].

The increased vascular diameter and MAP after VM seemed to have a major influence on the PWV and stiffness after low-pressure VM. A decrease in PWV was seen due to an increase in vascular diameter and BP after VM (Table 2). In particular, a significant correlation was observed between ΔVCD and both ΔPWV and Δstiffness. The VCD substantially affected the stiffness. The greater the elasticity of blood vessels, the greater their dilatability, resulting in an increased diameter of blood vessels [43]. In other words, an increased VCD subsequent to VM indicates an increased capacity for blood vessel dilation, thereby leading to decreased stiffness. Consequently, there appears to be a notable positive correlation between the increasing rate of VCD and the decreasing rate of the PWV and stiffness. Hence, the augmentation of VCD significantly contributes to the reduction of PWV and stiffness.

A decreased in the PWV and stiffness were also significantly associated with an increase in the MAP. During the initial VM performance, an increased MAP leads to an increase in vascular tone, which is the autoregulation of the myogenic response, and this vascular tone decreases at the end of the strain [44]. This response protects the blood vessels from hyper perfusion injury. Thus, decreased arterial pressure may release vascular tone and dilate the vessel. Moreover, increased vascular pulsatility appeared during phase III rather than during phase I of the VM [20]. The parasympathetic effects induced in the final phase of the VM help dilate the vessels. Therefore, vascular elasticity may improve after low-pressure VM.

Consequently, VM can increase BP and stimulate dynamic vascular function. This stimulation indicated that the increase in BP by the VM reduced stiffness and improved vascular distension. This demonstrates the possibility of controlling the PWV and stiffness on purpose. These results suggest that low-pressure VM contributes to changes in vascular dynamic function and positively affects vascular elasticity. Therefore, low-pressure VM activates the regulatory system of arterial circulation, which can lead to the dilation of blood vessels and reduce stiffness. This suggests that it may be a suitable method for improving vascular elasticity.

Since the experiment was conducted in healthy young adults, it is necessary to verify its effectiveness in patients of various ages. Additionally, there is a limitation in that there may be individual differences in expiratory pressure. All participants had expiratory pressures > 30 mmHg, but there was a clear difference in the capacity of expiratory pressure between males and females (Table 1). This difference may cause differences in biosignals and stiffness. Therefore, in future studies, it is necessary to determine the optimal stimulation pressure tailored to each individual considering not only sex but also individual respiratory ability, together with a device that can more delicately control expiratory pressure.

## Conclusion

In this study, low-pressure VM was applied with a focus on vascular stimulation rather than on respiratory capacity, and the effect of VM on vascular dynamic function was investigated. Low-pressure VM significantly increased the VCD, VDD, MAP, and DBP of the CCA and significantly decreased the stiffness and PWV. These results demonstrate that low-pressure VM is sufficient to improve blood vessel hemodynamics.

Performing optimized VM for vascular stimulation can be expected to lower the incidence of cerebrovascular diseases. This non-invasive treatment and prevention method can benefit a wide range of individuals, including healthy people as well as people with cardiovascular diseases, such as arteriosclerosis. With this method, various prospects can be expected, including the effects of reducing medical costs.

## Supporting information

S1 Checklist TREND statement checklist. (DOC)
